# Comparative Evaluation of Diagnostic Tools for Oxidative Deterioration of Polyunsaturated Fatty Acid-Enriched Infant Formulas during Storage

**DOI:** 10.3390/foods3010030

**Published:** 2013-12-27

**Authors:** Caroline Siefarth, Yvonne Serfert, Stephan Drusch, Andrea Buettner

**Affiliations:** 1 Department of Chemistry and Pharmacy, Emil Fischer Centre, Friedrich-Alexander University of Erlangen-Nürnberg, Schuhstr. 19, Erlangen 91052, Germany; E-Mail: caroline.siefarth@fau.de; 2 Fraunhofer Institute for Process Engineering and Packaging (IVV), Giggenhauser Str. 35, Freising 85354, Germany; 3 Department of Food Technology, University of Kiel, Heinrich-Hecht-Platz 10, Kiel 24118, Germany; E-Mail: yserfert@foodtech.uni-kiel.de; 4 Department of Food Technology and Food Material Science, Institute of Food Technology and Food Chemistry, Technical University of Berlin, Königin-Luise-Str. 22, Berlin 14195, Germany; E-Mail: stephan.drusch@tu-berlin.de

**Keywords:** antioxidants, autoxidation, infant formula, mineral composition, two-dimensional GC-MS, off-flavor, PUFA

## Abstract

The challenge in the development of infant formulas enriched with polyunsaturated fatty acids (PUFAs) is to meet the consumers’ expectations with regard to high nutritional and sensory value. In particular, PUFAs may be prone to fatty acid oxidation that can generate potential rancid, metallic and/or fishy off-flavors. Although such off-flavors pose no health risk, they can nevertheless lead to rejection of products by consumers. Thus, monitoring autoxidation at its early stages is of great importance and finding a suitable analytical tool to perform these evaluations is therefore of high interest in quality monitoring. Two formulations of infant formulas were varied systematically in their mineral composition and their presence of antioxidants to produce 18 model formulas. All models were aged under controlled conditions and their oxidative deterioration was monitored. A quantitative study was performed on seven characteristic odor-active secondary oxidation products in the formulations via two-dimensional high resolution gas chromatography-mass spectrometry/olfactometry (2D-HRGC-MS/O). The sensitivity of the multi-dimensional GC-MS/O analysis was supported by two additional analytical tools for monitoring autoxidation, namely the analysis of lipid hydroperoxides and conjugated dienes. Furthermore, an aroma profile analysis (APA) was performed to reveal the presence and intensities of typical odor qualities generated in the course of fatty acid oxidation. The photometrical analyses of lipid hydroperoxides and conjugated dienes were found to be too insensitive for early indication of the development of sensory defects. By comparison, the 2D-HRGC-MS/O was capable of monitoring peroxidation of PUFAs at low *ppb*-level in its early stages. Thereby, it was possible to screen oxidative variances on the basis of such volatile markers already within eight weeks after production of the products, which is an earlier indication of oxidative deterioration than achievable via conventional methods. In detail, oxidative variances between the formulations revealed that lipid oxidation was low when copper was administered in an encapsulated form and when antioxidants (vitamin E, ascorbyl palmitate) were present.

## 1. Introduction

Polyunsaturated fatty acids (PUFAs) may be easily oxidized to hydroperoxides via light-induced photooxygenation and/or in conjunction with further autoxidation processes. Thereby, radicals are formed in the presence of transition metals or heme proteins by a uni-molecular decomposition of hydroperoxides. Hence, peroxidation is initiated and then accelerates auto-catalytically. Several factors can promote autoxidation processes: fatty acid composition, oxygen partial pressure, concentration and activity of pro-oxidants and anti-oxidants, surface area of the product, and conditions of storage in terms of temperature, light, or water activity [1,2]. Infant formulas are high in PUFAs to match the fatty acid composition of mothers’ milk [3]. Thus, these products may be susceptible to fatty acid oxidation. Powdered infant formulas with a low water-activity and a large particle surface area are especially particularly susceptible to autoxidation. Furthermore, infant formulas are increasingly enriched with long chain (LC) *n*-3 and *n*-6 fatty acids [e.g., docosahexaenoic acid (DHA), arachidonic acid (AA), respectively] due to their purported promotion of visual and cognitive development [4].

Lipid oxidation leads to the formation of a broad variety of volatile and non-volatile substances. In several cases, the volatile substances formed are extremely odor-active. Thus, even small concentrations of typical secondary fatty acid oxidation products like aldehydes, ketones or alcohols can lead to rancid, fishy, metallic or cardboard-like off-flavors [5,6,7,8,9]. According to Grosch [10], unsaturated aldehydes with 6, 9 and 10 carbon atoms, respectively, and vinyl ketones with 8 carbon atoms are of special interest due to their high odor intensities and low odor thresholds, respectively [11]. Recently, a large number of odor-active volatile organic compounds (VOCs) were described as being responsible for a fishy off-odor profile in encapsulated fish oil products or formed in human milk during (freeze-) storage [5,12]. The volatile composition of infant formulas has also been previously studied [13,14]. Aldehydes, ketones, short chain fatty acids, sulfides and furanes, which are derived mainly from lipid autoxidation and thermal oxidation, could be identified via headspace sampling and GC-MS analyses. Many of these VOCs match those that were described as key contributors to fishy off-odor profiles, e.g., in stored human milk. Since the most recent study on the volatile profiles of infant formulas [13], comprehensive research has been undertaken and further advancements have been achieved, such as the enrichment of infant formulas with LC-PUFAs rather than replacing the milk fat with plain vegetable oils. As a drawback of such developments, sensory problems in enriched infant formulas might arise due to the increased susceptibility of the LC-PUFA-enriched matrices to lipid autoxidation. To the best of our knowledge, a combined analytical study of non-volatile primary products and odor-active secondary products of lipid autoxidation utilizing different analytical strategies, with additional aroma profile analysis (APA) of varying formulations of LC-PUFA-enriched infant formulas has not been reported in literature before. Accordingly, the aim of the present study was to identify the most sensitive method for monitoring lipid oxidation from its early stages for quality control purposes.

So far, standard tools for monitoring fatty acid oxidation are the determination of lipid hydroperoxides or peroxide value (PV) and conjugated dienes. In these methods, the formation of primary lipid oxidation products is measured either photometrically or iodometrically. A good overview of common methods for analysis of total hydroperoxides is given by Dobarganes and Velasco [15] with special focus on sensitivity and applications. The latter aspect is of great importance, especially when complex food matrices like infant formula emulsions are analyzed for lipid oxidation instead of pure fats and oils. However, an important aspect that must be kept in mind is that these primary oxidation products are just intermediates and prone to decomposition. Thus, another possibility is to measure the volatile, non-volatile and/or polymeric secondary oxidation products that are formed through decomposition reactions of hydroperoxides. With regard to lipid oxidation monitoring, the detection of VOCs and especially individual carbonyl compounds is gaining momentum in addition to the commonly established determination of total carbonyl content. For example, pentanal and hexanal are typically formed during the oxidation of *n*-6 PUFAs, and propanal is known as a characteristic secondary oxidation product of *n*-3 PUFAs [16]. In general, hexanal analysis via headspace GC methods or GC-MS is well established in the food industry as an indicator for lipid oxidation [17,18]. The advantage of GC-MS compared to most common analytical methods used for carbonyl determinations (e.g., spectrophotometry of chromophoric derivatives or combined with high-performance liquid chromatography, HPLC) is its high sensitivity in combination with mass specificity. Furthermore, the accuracy and precision of GC-MS can be further improved when used in combination with stable isotope dilution assays (SIDA) as well as combinatory or multi-dimensional approaches such as GC-GC (heart-cut)-MS [19].

In the present study, three different methods for monitoring lipid oxidation were comparatively applied to 18 LC-PUFA-enriched and spray-dried infant formulas that were formulated to provide a range of combinatory compositions of minerals and antioxidants. A range of odor-active secondary oxidation marker substances were monitored in the formulas by means of 2D-HRGC-MS in combination with SIDA over a storage period of two months. Lipid hydroperoxides were determined in parallel for a storage period of up to one year via the commonly established thiocyanate method, as well as conjugated dienes by conventional UV detection (established protocols, *cf.* Section 2.7).

Quantitative data on odor-active compounds obtained from GC-MS analyses as well as the two standard oxidation parameters were compared with the results of an APA on typical odor qualities of the *n*-3 and *n*-6 fatty acid enriched infant formulas. In addition, a trained sensory panel evaluated if the observed flavor changes were rated as off-flavor in the respective infant formulas.

## 2. Experimental Section

### 2.1. Materials

The powdered infant formulas consisted of the following ingredients (supplier in parentheses): skim milk (obtained locally from a milk processing company, Cremilk GmbH, Kappeln, Germany), skim milk powder (Ledor MMP-S UST, Hochdorf Swiss Milk AG, Hochdorf, Switzerland), whey protein concentrate (WPC P45, Prolactal GmbH, Hartberg, Austria), vegetable fats and oils (palm, rapeseed, linseed, coconut, sunflower, high oleic sunflower) (Florin AG, Muttenz, Switzerland), butter (Emmi Schweiz AG, Luzern, Switzerland), maltodextrin (Tereos Syral S.A.S., Marckolsheim, France), lactose (Milei GmbH, Leutkirch, Germany), dietary fibers: galacto-oligosaccharides (GOS, Vivinal^®^ GOS, FrieslandCampina Domo EMEA, Amersfoort, the Netherlands) and fructo-oligosaccharides (FOS, Selectchemie AG, Zürich, Switzerland), soya lecithin (Solae Europe S.A., Le Grand-Saconnex, Switzerland), monoglycerides based on rapeseed and palm oil (Dimodan^®^ PH 100 NS/B Kosher, DuPont™ Danisco^®^, Danisco Switzerland AG, Switzerland), fish oil (Marinol D-40, Stepan Lipid Nutrition Europe, Koog aan de Zaan, the Netherlands), arachidonic acid oil (Martek Biosciences, DSM Nutritional Products Europe Ltd., Basel, Switzerland), nucleotides (DSM Nutritional Products Europe Ltd., Heerlen, the Netherlands), lactoferrin (Morinaga Milk Industry Co. Ltd., Tokyo, Japan), antioxidants ascorbyl palmitate and dl-α-tocopherol (Ronoxan^®^ A, DSM Nutritional Products Europe Ltd.), vitamins and minerals. A mixture of vitamins and semi-essential nutrients [vitamin C (sodium ascorbate), vitamin E, vitamin A, niacin, vitamin D, pantothenic acid, vitamin K, vitamin B12, biotin, vitamin B1, folic acid, vitamin B6, vitamin B2, choline bitartrate, taurine, inositol, l-carnitine] was purchased from Glanbia Nutritionals Deutschland GmbH (Orsingen-Nenzingen, Germany). Mineral supplements were also purchased as a single mixture (magnesium chloride, manganese sulfate, sodium selenite, zinc sulfate and potassium iodate) from Glanbia Nutritionals Deutschland GmbH. The copper and iron compositions of the infant formulas were varied over 18 different samples. Copper was added either as Cu(II)-sulfate (Glanbia Nutritionals Deutschland GmbH), encapsulated Cu(II)-sulfate (Vitablend Nederland B.V., Wolvega, the Netherlands) or Cu-lysine-complex. The latter complex was prepared prior to the experiments from Cu(II)-sulfate and l-lysine HCl (Selectchemie AG). Iron was added to the formulas either as Fe(II)-gluconate (Gluconal^®^, Purac Biochem, Gorinchem, the Netherlands), Fe(III)-pyrophosphate (LipoFer^®^, Lipofoods, Barcelona, Spain) or encapsulated Fe(II)-sulfate (Vitablend Nederland B.V.).

An overview of the formula composition in relation to the variations of copper and iron is given in Section 2.3.

### 2.2. Chemicals

GC-MS analysis: The reference compounds with the stated purities were as follows (supplier in parentheses): hexanal 98%, (*E*,*Z*)-nona-2,6-dienal 95%, oct-1-en-3-one 50% (Aldrich, Steinheim, Germany), (*E*,*E*)-deca-2,4-dienal 85%, (*E*)-hex-2-enal 97% (Fluka, Steinheim, Germany), (*Z*)-octa-1,5-dien-3-one 99%, *trans*-4,5-epoxy-(*E*)-dec-2-enal 90% (aromaLAB AG, Freising, Germany). The following stable isotope labeled standards were additionally purchased from aromaLAB: [5,5,6,6-^2^H_4_]-hexanal, [1,2-^13^C_2_]-(*E*,*Z*)-nona-2,6-dienal, [1,2-^2^H_2_-3]-oct-1-en-3-one, [4,5-^2^H_2_]-(E,E)-deca-2,4-dienal, [2,3-^2^H_2_]-(E)-hex-2-enal, [5,6-^2^H_2_]-(Z)-octa-1,5-dien-3-one, [9,9,10,10-^2^H_4_]-trans-4,5-epoxy-(E)-dec-2-enal. Dichloromethane p.a. was used as the organic solvent and extracting agent (Th. Geyer GmbH and Co. KG, Renningen, Germany). Anhydrous sodium sulfate was purchased from chemsolute^®^ (Th. Geyer GmbH and Co. KG, Renningen, Germany).

Thiocyanate assay and analysis of conjugated dienes—the following chemicals were used—ammonium thiocyanate (Sigma Aldrich, St. Louis, MO, USA), barium chloride, iron-(II)-sulphate heptahydrate, isooctane (Carl Roth GmbH and Co. KG, Karlsruhe, Germany), 2-propanol (VWR International, Pty Ltd., Murarrie, Australia).

### 2.3. Experimental Design

Manufactured infant formulas varied in the use of antioxidants (ascorbyl palmitate and dl-α-tocopherol), as well as in copper and iron composition. Either an encapsulated variant of the minerals or a non-encapsulated alternative was used. In the non-encapsulated case, iron variants with different valences (Fe^2+^, Fe^3+^) and/or copper variants (Cu^2+^ and a Cu-lysine-complex) were processed (*cf.* Section 2.1). An overview of the 18 different formulations is given in Table 1.

**Table 1:** Formulations of the manufactured infant formulas with respect to the presence of antioxidants (ascorbyl palmitate and dl-α-tocopherol) and mineral composition in relation to copper and iron (*n*_formulations_ = 18). X: used ingredient(s).

Formulation	Antioxidants	Cu(II)-Sulfate	Cu-Lysine-Complex	Cu(II)-Sulfate, Encapsulated	Fe(II)-Gluconate	Fe(III)-Pyrophosphate	Fe(II)-Sulfate, Encapsulated
A1	X	X	-	-	X	-	-
A2	X	X	-	-	-	X	-
A3	X	X	-	-	-	-	X
A4	X	-	X	-	X	-	-
A5	X	-	X	-	-	X	-
A6	X	-	X	-	-	-	X
A7	X	-	-	X	X	-	-
A8	X	-	-	X	-	X	-
A9	X	-	-	X	-	-	X
B1	-	X	-	-	X	-	-
B2	-	X	-	-	-	X	-
B3	-	X	-	-	-	-	X
B4	-	-	X	-	X	-	-
B5	-	-	X	-	-	X	-
B6	-	-	X	-	-	-	X
B7	-	-	-	X	X	-	-
B8	-	-	-	X	-	X	-
B9	-	-	-	X	-	-	X

### 2.4. Manufacturing of Infant Formula

The primary materials for producing infant formulas were mixed in several steps. First of all, fats and oils were heated to 63 °C. Therein, antioxidants and emulsifiers were dissolved, as well as fish oil and arachidonic acid oil. Skim milk was heated to 55 °C and skim milk powder was dissolved in the skim milk. All ingredients were added step-by-step. In a final step, the fat mix was added and the suspension was pre-dispersed using a rotor-stator-blender (Ystral GmbH, Ballrechten-Dottingen, Germany) at 16,000 rpm for 5 min. Subsequently, a two-step homogenization was performed (Panda 2K, Niro Soavi Deutschland, Lübeck, Germany) at 150/50 bar. The formula milk emulsion was spray-dried at a dry substance content of 50% (Mobile Minor, Niro Inc., Copenhagen, Denmark) with two-fluid nozzle at 170 °C/80 °C inlet and outlet temperature, respectively. After spray-drying, lactoferrin was added under dry conditions. The combined formula milk powders were packaged in aluminum bags under 20% vacuum (VM-19/S/CL, Röscher GmbH, Berlin, Germany). The packaged samples were stored unopened at constant temperature of 21 ± 1 °C for up to 12 months. The powdered infant formulas were analyzed for lipid oxidation products immediately after manufacturing, as well as after the following storage periods (months): 1, 2, 3, 6, 9 and 12 at room temperature.

### 2.5. Sample Preparation

For analyses of lipid oxidation products, the powdered formula milks were reconstituted with tap water as follows: 180 mL of tap water was boiled in an Erlenmeyer flask for 1 min and cooled down to 40 °C. Formula milk powder (27.0 ± 0.1 g) was weighed in a beaker and the tap water was added. The mixture was shaken well for 1 min.

For GC-MS analyses, the reconstituted infant formulas were first subjected to solvent assisted flavor evaporation (SAFE). This distillation technique is known for its fast and gentle isolation of volatiles from food matrices [20]. At room temperature (21 ± 1 °C), 25.0 (±0.1) g formula milk was combined with freshly-distilled dichloromethane at a ratio of 2:1 (milk:solvent). Additionally, 1 mL of labeled internal standards was added to the mixture. Concentrations of seven characteristic odor-active secondary oxidation products (see Section 2.2) were determined in a formula milk reference in preliminary experiments and comparable amounts were added as isotope labeled internal standard to the milk-solvent mixture for SIDA. The combined mixture was stirred for 30 min, after which it was immediately distilled (high vacuum conditions, 50 °C water bath temperature and 55 °C water temperature within the SAFE apparatus). After distillation, additional aliquots of 10 mL of dichloromethane were administered to the residue and distillation was re-performed. The latter step was performed to achieve a complete transfer of the VOCs from the milk matrix residue to the distillate. The organic phase of the distillate was separated and the aqueous phase was twice extracted with 15 mL of dichloromethane. Subsequently, the combined organic phases were dried with anhydrous sodium sulfate, filtered and concentrated to a total volume of 100–200 μL via Vigreux-distillation and micro-distillation at 50 °C [21].

For GC-MS analysis of the powdered infant formulas immediately after production, three independent reconstitutions of the 18 formulations were performed and prepared for GC-injection, as described above. For further GC-MS analyses of lipid oxidation products, three independent reconstitutions were randomly performed for one third of the samples (six formulations).

For analysis of lipid hydroperoxides and conjugated dienes, formula powders were dissolved in distilled water and extracted with isooctane/2-propanol, with 7 mL of the isooctane/2-propanol mixture (1:1, v/v) added to 2.5 mL of the sample. The mixture was stirred for 30 s, centrifuged at a relative centrifugal force of 720× *g* (Allegra 2IR Centrifuge, Beckmann Coulter, Brea, CA, USA), and the organic phase was used for lipid isolation. An aliquot of the organic phase was transferred to a test tube and the organic solvent was removed under a stream of nitrogen. Analyses were performed in triplicate on two independent reconstitutions of powdered formulas.

### 2.6. Quantification by Stable Isotope Dilution Assays (SIDA)

Seven selected odorants known as secondary fatty acid oxidation markers (see Section 2.2) were quantified in the infant formulas directly after production, as well as after four and eight weeks of storage, respectively. The selection was based on a previous GC/O screening on about 20 infant formula samples with a fishy off-flavor (results not shown). Compared to a reference, several odor impressions were detected in the off-flavor samples, which could be identified by comparison with reference substances based on the following criteria: odor quality, retention indices on two stationary phases and mass spectra. Identification and quantification were performed using a two-dimensional gas chromatographic system (2D-HRGC-MS/O) which consisted of two helium CP 3800 GCs (Varian Inc., Darmstadt, Germany) in combination with a Saturn 2200 MS (Varian Inc.) and sniffing ports for olfactory detection.

Concentrated distillates (2 μL) containing the target volatiles and labeled internal standards were injected into the GC system using the cold-on-column technique at 40 °C. Injections were performed in triplicate. The 2D-HRGC-MS measurements were executed using the following capillaries: DB-FFAP (30 m × 0.32 mm fused silica capillary, free fatty acid phase FFAP, 0.25 μm; type Chrompack, Varian) in the first oven and DB-5 (30 m × 0.25 mm fused silica capillary DB-5, 1.5 μm; type J and W, Agilent Technologies, Santa Clara, CA, USA) in the second oven. The oven programs were as follows: after 2 min, the oven temperature was raised to 240 °C (DB-FFAP) and 250 °C (DB-5), respectively, at a rate of 10 °C·min^−1^. The final temperatures were held for 5 min. The flow rate of the helium carrier gas was 2.5 mL·min^−1^. The effluent was split at the end of the columns using deactivated and uncoated fused silica capillaries (100 cm × 0.2 mm). In the first oven, the effluent was split into a sniffing port and a flame ionization detector (FID). A specific section of the effluent, containing the volatile and its labeled standard (retention time ± 0.2 min) was transferred via a cryo trap to the second oven. At the end of the second oven, the effluent was split into a second sniffing port and the MS. The FID detector was set to 250 °C and the sniffing ports to 300 °C. Mass spectra in the chemical ionization mode (MS/CI) were acquired with methanol as the reagent gas at a flow of 2.5 mL·min^−1^. Selected ions of the odorants and labeled standards were analyzed in scan mode (*m/z* 60–249 range) and their intensities were calculated by means of Varian MS-Workstation, MS Data Review (Version 6.9; Service Pack 1, Varian Inc.).

Concentrations were calculated by means of calibration data obtained by measuring defined mixture ratios of respective labeled and unlabeled compounds. Furthermore, MS response factors were determined by measuring defined mixtures of respective labeled and unlabeled compounds. Further details on quantification, e.g., selected ions or calibration factors, are given in Table 2.

**Table 2:** Odorants, selected ions for quantification by stable isotope dilution assays and details on calibration factors determined on film capillary DB-5. *R*^2^—coefficient of determination.

Odorant	Ion (*m/z*)	Internal Standard	Ion (*m/z*)	Calibration Line	*R*^2^
hexanal	83	[5,5,6,6-^2^H_4_]-hexanal	87	*y* = 0.4915*x* − 0.0475 ^1^	0.9853
				*y* = 0.6059*x* − 0.0690 ^2^	0.9958
(*E*)-hex-2-enal	99	[2,3-^2^H_2_]-(*E*)-hex-2-enal	101	*y* = 0.6555*x* + 0.0538 ^1^	0.9999
				*y* = 0.8132*x* − 0.1313 ^2^	0.9984
(*E*,*Z*)-nona-2,6-dienal	121	[1,2-^13^C_2_]-(*E*,*Z*)-nona-2,6-dienal	123	*y* = 0.8522*x* + 0.0784 ^1^	0.9996
				*y* = 1.0200*x* − 0.0600 ^2^	0.9938
(*Z*)-octa-1,5-dien-3-one	125	[5,6-^2^H_2_]-(*Z*)-octa-1,5-dien-3-one	127	*y* = 0.9355*x* − 0.0323	0.9998
oct-1-en-3-one	127	[1,2-^2^H_2-3_]-oct-1-en-3-one	129–130	*y* = 1.2092*x* − 0.0408 ^1^	0.9970
				*y* = 1.6112*x* − 0.1673 ^2^	0.9989
(*E*,*E*)-deca-2,4-dienal	153	[4,5-^2^H_2_]-(*E*,*E*)-deca-2,4-dienal	155	*y* = 0.7705*x* − 0.1005 ^1^	0.9952
				*y* = 0.8218*x* − 0.0851 ^2^	0.9988
*trans*-4,5-epoxy-(*E*)-dec-2-enal	153	[9,9,10,10-^2^H_4_]-*trans*-4,5-epoxy-(*E*)-dec-2-enal	157	*y* = 1.4603*x* − 0.0139	0.9998

^1^ calibration factors used for odorants at low *ppb*-level concentrations; ^2^ calibration factors used for odorants at high *ppb*-level concentrations.

### 2.7. Determination of Lipid Hydroperoxides and Conjugated Dienes

The determination of hydroperoxides and conjugated dienes was performed immediately after formula production and additionally at selected times up to 12 months of storage (*cf.* Section 2.4). The sample preparation is described in Section 2.5. After reconstitution of the powders with distilled water, lipids were extracted from the infant formulas using a blend of ethanol, hexane, and ethyl acetate, as described by Satué-Gracia [22].

Hydroperoxides: the International Dairy Federation standard method [23], as described in Drusch *et al.* [24] with slight modifications, was used for the determination of the hydroperoxide content. This photometrical assay is based on the oxidation of Fe(II) to Fe(III) ions that form a colored complex with ammonium thiocyanate. The extinction coefficient was measured at 485 nm.

Conjugated dienes: after dilution of the organic phase of the lipid extractions with 2-propanol, conjugated dienes were determined photometrically at 234 nm. For calculation of the concentration, the results were expressed as millimoles of hydroperoxides per kg fat using a molar coefficient of 26,000 for methyl linoleate hydroperoxides [25].

### 2.8. Aroma Profile Analysis (APA)

A volume of 20 mL of the respective reconstituted formula milk was filled into sensory glass beakers (140 mL, J. Weck GmbH u. Co. KG, Wehr, Germany) and closed with a lid. Sensory analyses were performed in a sensory panel room at 21 ± 1 °C. Trained panelists (*n* = 8–12, male/female, age 24–45) from the University of Erlangen-Nuremberg (Erlangen, Germany) and Fraunhofer IVV (Freising, Germany) with normal olfactory and gustatory function participated in the APA sessions and exhibited no known illness at the time of examination. Prior to this study, the assessors were recruited in weekly training sessions for the recognition of about 100 selected odor-active compounds according to their odor qualities by means of an in-house developed flavor language.

In a first session the panelists were asked to orthonasally evaluate the samples and the named odor attributes of the formula milks were collected. Attributes that were detected by more than 50% of the panelists were selected for subsequent evaluations. In subsequent sessions, the panelists were asked to score the orthonasally perceived intensities of the selected attributes on a seven-point-scale from 0 (no perception) to 3 (strong perception) in increments of 0.5. The order of presentation of the infant formulas was randomized and no information on the purpose of the experiment or the composition of the samples was given to the panelist. Aroma profile analysis was performed on samples immediately after production, as well as after four, 8 and 24 weeks (six months) of storage. The results for each odor attribute and sample were averaged and plotted in a box-plot diagram.

In addition to the APA, the panelists were asked if they perceived an off-flavor in the respective infant formulas. When an off-flavor was recorded, rating of the overall odor impression of the products was based on a yes/no answer.

To check changes in color over storage period, an instrumental color analysis was carried out using a Chroma Meter CR-300 (Konika Minolta Inc., Marunouchi, Japan) with a DP-301 data processor. Calibration was performed on a white standard (CR-A43, Konika Minolta Inc.). The color was expressed in L*a*b* mode, in which L* represents the lightness value, and a* and b* values the chromaticity coordinates. The samples were analyzed in triplicate.

### 2.9. Statistical Analyses

For statistical analysis, Student’s *t*-tests (or Welch-tests), Mann-Whitney *U*-tests and one-way repeated analysis of variances (repeated ANOVA) were carried out (as declared within the respective sections of the manuscript). In the latter case, either the Tukey HSD or the Fisher LSD procedure was used to detect significant differences between specific storage times. Statistical analyses were performed using OriginPro 9G (OriginLab Co., Northampton, MA, USA) and Statistica 10 (StatSoft Europe GmbH, Hamburg, Germany) software, respectively. For all analyses, the level of statistical significance was set at 5%. Outliers were identified as individual values that were not within two standard deviations of the preceding and succeeding storage time data points.

## 3. Results

### 3.1. Monitoring of Autoxidation—Quantification of Secondary Lipid Oxidation Products by Means of 2D-HRGC-MS/O

Seven representative compounds of secondary lipid oxidation reactions were chosen among the alkanal, alkenal and alkadienal substance classes, as well as alkenones and alkadienones, which are predominantly formed during autoxidation. According to the literature, these odorants have previously been identified in infant formulas and human breast milk, respectively, since both mothers’ milk and infant formulas are rich in PUFAs [5,13]. Furthermore, these compounds were also identified in *n*-3 fatty acid enriched fish oil capsules that are administered during pregnancy and the lactation period to supplement the mothers’ nutritional fatty acid level [26]. Table 3 shows the quantitative data of the selected odorants in infant formulas directly after production (week 0) supplemented with antioxidants (formulations A1–A9) and without supplementation of antioxidants (formulations B1–B9). All compounds were present in the formula milks in the lower μg·kg^−1^ (*ppb*) range, with (*E*)-hex-2-enal, (*Z*)-octa-1,5-dien-3-one and (*E*,*Z*)-nona-2,6-dienal exhibiting the lowest concentrations below 2 μg·kg^−1^. Oct-1-en-3-one and (*E*,*E*)-deca-2,4-dienal were detected in the freshly produced formulations at concentrations below 10 μg·kg^−1^. *trans*-4,5-Epoxy-(*E*)-dec-2-enal was determined at concentrations of up to 20 μg·kg^−1^ and hexanal at concentrations of between 5 and 50 μg·kg^−1^.

**Table 3:** Quantification of selected odorants in infant formulations directly after production (with and without antioxidants).

Formulation	Concentration (μg·kg^−1^)													
	Hexanal		(*E*)-Hex-2-enal		Oct-1-en-3-one		(*Z*)-Octa-1,5-dien-3-one		(*E*,*Z*)-Nona-2,6-dienal		(*E*,*E*)-Deca-2,4-dienal		*trans*-4,5-Epoxy-(*E*)-dec-2-enal	
	Mean	SD	Mean	SD	Mean	SD	Mean	SD	Mean	SD	Mean	SD	Mean	SD
A1	10.93	±1.85	0.22	±0.14	3.43	±0.49	0.05 ^a^	±0.00	0.43 ^a^	±0.00	0.99	±0.32	4.22	±0.81
A2	5.73 ^a^	±1.24	0.09	±0.01	3.42	±0.25	0.05 ^a^	±0.01	0.32 ^a^	±0.08	3.07	±0.71	9.66	±0.88
A3	9.87	±2.77	0.20	±0.14	4.02	±0.85	0.06 ^a^	±0.06	0.48 ^a^	±0.15	2.57	±0.35	6.50	±4.27
A4	14.4	− *	0.13	− *	3.62	− *	<0.01 ^a^	− *	<0.01 ^a^	− *	4.21	− *	5.49	− *
A5	10.60 ^a^	− *	<0.01 ^a^	− *	3.23	− *	0.03 ^a^	− *	<0.01 ^a^	− *	4.60	− *	14.79	− *
A6	10.11 ^a^	− *	<0.01 ^a^	− *	3.22	− *	0.02 ^a^	− *	<0.01 ^a^	− *	6.20	− *	15.33	− *
A7	6.23 ^a^	±0.73	0.12 ^a^	±0.04	2.46	±0.39	<0.01 ^a^	±0.01	0.24 ^a^	±0.03	3.17	±0.20	5.61	±1.03
A8	8.35 ^a^	±4.90	0.04 ^a^	±0.03	1.97	±0.22	0.02 ^a^	±0.01	0.25 ^a^	±0.09	1.92	±0.34	3.44	±0.30
A9	9.68	±3.78	0.17	±0.17	2.61	±0.55	0.01 ^a^	±0.01	0.23 ^a^	±0.15	2.47	±0.42	6.80	±0.83
B1	35.96	±4.10	0.32	±0.02	1.52	±0.17	0.15	±0.05	1.28	±0.12	3.83	±0.36	9.69	±0.24
B2	36.49	±7.45	0.49	±0.28	1.81	±0.57	0.07 ^a^	±0.01	0.60	±0.20	3.50	±2.71	6.53	±0.44
B3	30.81	±8.73	0.21	±0.01	2.14	±0.03	0.01 ^a^	±0.01	0.79	±0.06	2.56	±0.35	9.83	±0.87
B4	33.42	±5.34	0.22	±0.04	1.69	±0.12	0.01 ^a^	±0.01	0.61	±0.08	4.14	±1.55	10.25	±2.13
B5	37.12	±9.34	0.16	±0.04	1.86	±0.12	0.01 ^a^	±0.01	0.64	±0.03	2.98	±0.40	9.73	±1.08
B6	41.11	±7.67	0.37	±0.21	2.16	±0.38	0.03 ^a^	±0.01	0.69	±0.19	2.93	±0.26	11.74	±2.13
B7	37.99	±6.71	0.66	− *	2.04	±0.46	0.03 ^a^	±0.03	0.72	±0.12	3.88	±0.22	7.50	±1.72
B8	30.43	±10.13	0.42	±0.11	1.13	±0.05	< 0.01 ^a^	±0.00	0.63	±0.12	2.60	±0.78	6.16	±1.79
B9	33.82	±11.25	0.38	±0.19	1.44	±0.13	< 0.01 ^a^	±0.01	0.72	±0.19	2.54	±0.23	10.00	±1.44

Reconstitution of powdered formulas was performed in triplicate. * reconstitution of powdered formulas was not performed in triplicate; ^a^ determinations yielding values below the linearity of the calibration curve (limit of quantification, LOQ).

**Figure 1:**
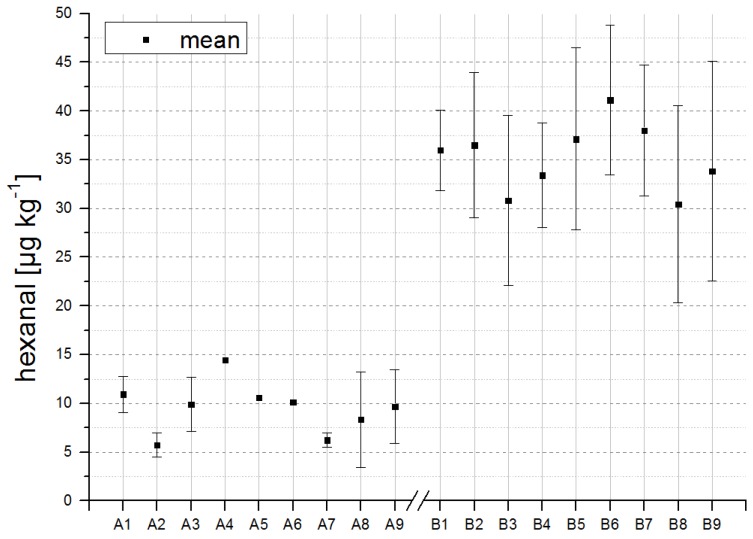
Overview of hexanal concentrations in formulations with antioxidants (**A**) and without antioxidants (**B**). Reconstitution of powdered formulas was performed in triplicate.

The detection of low concentrations of the respective odorants in freshly produced infant formulas demonstrates that autoxidation is already initialized during production. For some compounds, differences seemed to arise in the concentrations of A-formulations (with antioxidants) and B-formulations (without antioxidants). To validate the significance of such differences between the formulations with and without antioxidants, a Student’s *t*-test, Welch-test or Mann-Whitney *U*-test was performed, depending on normal distribution and homogeneity of variances of the data (Table 4). Differences between A-formulations and B-formulations were significant in four out of seven secondary lipid oxidation markers [hexanal, (*E*)-hex-2-enal, oct-1-en-3-one, (*E*,*Z*)-nona-2,6-dienal]. Details on statistical analyses are given in Table 4. The most significant changes were observed for hexanal, as shown in Figure 1. Thus, differences between A- and B-formulations, as shown for hexanal in Figure 1, are detectable by means of multi-dimensional GC-MS even directly after production, highlighting the high sensitivity of this method.

**Table 4:** Statistical data on comparison of means between formulations with and without antioxidants (week 0). *t* and |*Z*| as critical values of the respective statistical test.

Odorant	Comparison of Means		
	*t*(16), |*Z*|	*Significance* (*p* < 0.05)	*p*-Value
hexanal	−17.79 ^a^	sig.	5.77 × 10^−12^
(*E*)-hex-2-enal	−4.26 ^a^	sig.	5.93 × 10^−4^
oct-1-en-3-one	5.58 ^a^	sig.	4.12 × 10^−5^
(*Z*)-octa-1,5-dien-3-one	0.26 ^c^	n.s.	0.79
(*E,Z*)-nona-2,6-dienal	−3.54 ^c^	sig.	4.01 × 10^−4^
(*E*,*E*)-deca-2,4-dienal	0.04 ^b^	n.s.	0.97
*trans*-4,5-epoxy-(*E*)-dec-2-enal	−0.67 ^b^	n.s.	0.52

^a^ Student’s *t*-test; ^b^ Welch-test; ^c^ Mann-Whitney *U-* test.

Table 5 and Table 6 summarize the results of quantification after four weeks and eight weeks of storage.

After four weeks, the selected odorants were still present in the infant formulas at concentrations in the lower *ppb*-range (below 10 μg·kg^−1^), with the exception of *trans*-4,5-epoxy-(*E*)-dec-2-enal and hexanal, which showed higher concentrations already directly after production. Nevertheless, the concentrations of the latter two odorants showed an increase compared to week 0 but were still below 100 μg·kg^−1^ after four weeks of storage. After eight weeks of storage, most formulations showed a (further) increase in the formation of secondary lipid oxidation products. Whereas (*E*)-hex-2-enal, (*Z*)-octa-1,5-dien-3-one and (*E*,*Z*)-nona-2,6-dienal stayed at concentrations below 10 μg·kg^−1^, the concentration of (*E*,*E*)-deca-2,4-dienal and oct-1-en-3-one increased in some cases up to 50 to 60 μg·kg^−1^. The highest increases in concentrations for some formulations were detected for hexanal and *trans*-4,5-epoxy-(*E*)-dec-2-enal, with concentrations of up to 850 μg·kg^−1^.

These observations on increasing concentrations of secondary lipid oxidation products with storage time confirm the on-going autoxidation processes in the packaged, evacuated infant formulas. Nevertheless, some formulations were obviously quite stable over storage time with respect to the formation of these lipid oxidation products (A1, A4–A6, A7–A9, B7–B8) as shown in Table 5 and Table 6, while other formulations (B1–B3, B4–B6, B9) were prone to considerable oxidative effects. The data set of the first eight weeks of storage confirms that A-formulations with antioxidants remained more stable than B-formulations without antioxidants, as demonstrated by the reference data of week 0. Table 7 details the statistical interpretation of the data, showing that differences between A- and B-formulations were significant after four weeks of storage in four out of seven secondary lipid oxidation markers [hexanal, (*E*)-hex-2-enal, (*E*,*Z*)-nona-2,6-dienal, *trans*-4,5-epoxy-(*E*)-dec-2-enal] and after eight weeks for all odorants under investigation.

Only two formulations without antioxidants were found to be stable: B7 and B8, respectively (Table 5 and Table 6). Thus, the mineral combination used in these formulations, which potentially acts as a pro-oxidant, seems to trigger less oxidation than other combinations on basis of the quantified volatile oxidation markers. In these two formulas, encapsulated Cu^2+^ was manufactured together with non-encapsulated Fe^2+/3+^. The equivalent formulas with antioxidants (A7 and A8) also remained very stable over a storage period of eight weeks.

**Figure 2:**
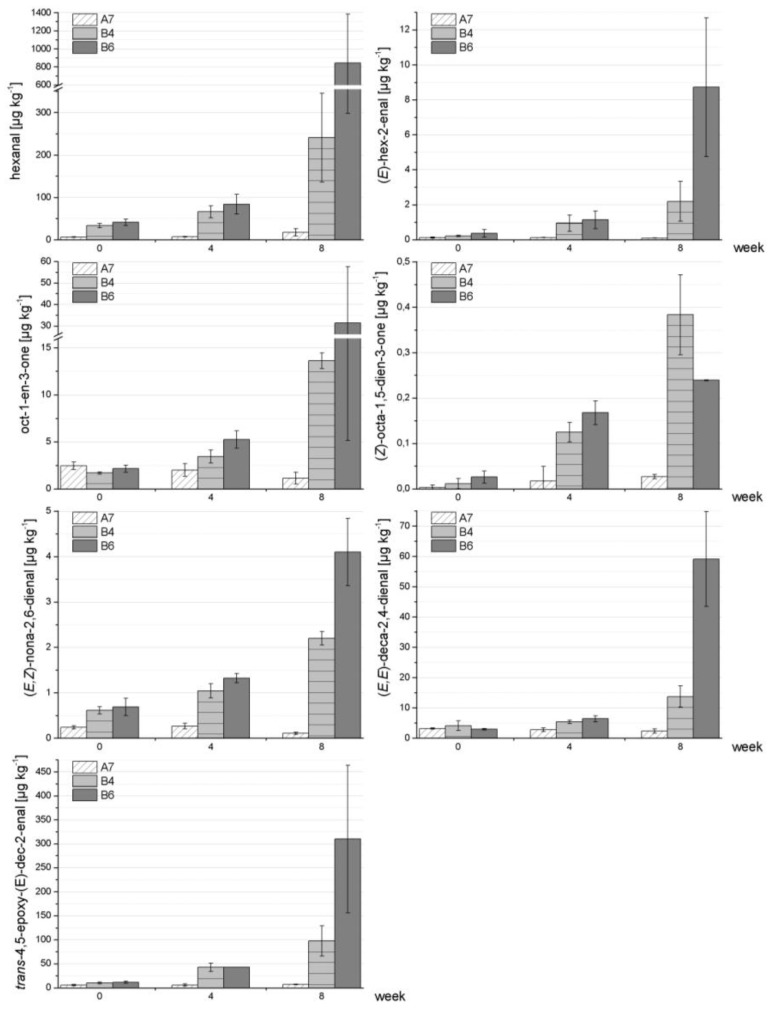
Example of a stable formulation (A7) in comparison to two less stable formulations (B4, B6) with respect to lipid autoxidation/formation of secondary lipid oxidation markers. Reconstitution of powdered formulas was performed in triplicate.

Figure 2 shows the data for a very stable formulation (A7) in comparison to two formulations that were found to be less stable (B4, B6). The plots clearly demonstrate that all chosen oxidation markers were well-suited to reveal differences in the stability of the respective formulations. Statistical analyses (Table 8) demonstrated the following: whereas in most cases a significant increase in the formation of such oxidation markers was observed for the less stable formulations B4 and B6 comparing weeks 0–8, no significant increase (or decrease) was found for the stable formulation A7 during the storage period of eight weeks [only exception: hexanal (weeks 0–8)]. The only exception in the B-formulations was B6 for oct-1-en-3-one (weeks 0–8).

**Table 5:** Quantification of selected odorants in infant formulations after four and eight weeks of storage with antioxidants.

Storage (Weeks)	Formulation	Concentration (µg·kg^−1^)						
		Hexanal	(*E*)-Hex-2-enal	Oct-1-en-3-one	(*Z*)-Octa-1,5-dien-3-one	(*E*,*Z*)-Nona-2,6-dienal	(*E*,*E*)-Deca-2,4-dienal	*trans*-4,5-Epoxy-(*E*)-dec-2-enal
4	A1	11.40 ^a^	0.11	1.83	<0.01 ^a^	0.47 ^a^	1.52	4.61
8		16.14	0.11	1.69	0.05 ^a^	0.47 ^a^	5.07	17.65
4	A2	20.97	0.33	5.33	0.18	0.87	5.71	14.94
8		43.54	0.73	6.37	0.46	2.37	3.78	64.84 ^a^
4	A3	20.32	0.23	5.22	0.21	0.91	6.56	24.37 ^a^
8		70.03	0.64	6.02	0.40	2.54	5.34	79.56 ^a^
4	A4	12.97	0.13	2.79	0.05 ^a^	0.51 ^a^	4.20	6.12
8		15.77	0.20	3.02	0.10 ^a^	0.64	3.19	8.62
4	A5	10.90 ^a^	0.09	2.11	0.04 ^a^	0.36 ^a^	3.28	5.21
8		16.70	0.23	5.90	ND	ND	3.24	18.11 ^a^
4	A6	14.99	0.18	3.23	0.05 ^a^	0.41 ^a^	3.95	11.95
8		24.93	0.30	3.61	0.10 ^a^	0.81	3.67	27.73 ^a^
4	A7	6.99 ^a^	0.13	2.01	0.05 ^a^	0.31 ^a^	2.79	5.87
8		17.56	0.09	1.14	0.02 ^a^	0.09 ^a^	2.36	6.95
4	A8	3.87 ^a^	0.04	1.39	<0.01 ^a^	0.25 ^a^	2.80	3.67
8		5.72 ^a^	0.08	2.21	0.07 ^a^	0.52 ^a^	7.65	47.55 ^a^
4	A9	6.02 ^a^	0.09	2.05	0.04 ^a^	0.39 ^a^	2.53	2.67
8		6.82 ^a^	0.06	2.32	0.02 ^a^	0.35 ^a^	3.23	6.29

^a^ Determinations yielding values below as well as above the linearity of the calibration curve (LOQ and upper LOQ, respectively); ND—not detected (below limit of detection, LOD).

**Table 6:** Quantification of selected odorants in infant formulations after four and eight weeks of storage without antioxidants.

Storage (Weeks)	Concentration (µg kg^−1^)							
	Formulation	Hexanal	(*E*)-Hex-2-enal	Oct-1-en-3-one	(*Z*)-Octa-1,5-dien-3-one	(*E*,*Z*)-Nona-2,6-dienal	(*E*,*E*)-Deca-2,4-dienal	*trans*-4,5-Epoxy-(*E*)-dec-2-enal
4	B1	41.34	0.44	2.01	0.25	2.28	5.56	26.09 ^a^
8		65.17	0.83	4.71	0.23	2.52	14.16	49.71 ^a^
4	B2	35.32	ND	2.62	ND	0.80	4.79	32.77 ^a^
8		103.89	1.01	8.38	0.31	2.21	8.69	106.53 ^a^
4	B3	53.06	0.69	5.38	0.18	1.69	4.15	8.60
8		259.79 ^a^	3.42 ^a^	37.76 ^a^	0.33	6.35	27.11 ^a^	320.51 ^a^
4	B4	65.83	0.95	3.45	0.12	1.04	5.41	42.46 ^a^
8		240.88 ^a^	2.19 ^a^	13.62 ^a^	0.38	2.20	13.74	97.51 ^a^
4	B5	50.24	0.57	6.07	0.16	1.55	5.48	92.97 ^a^
8		129.03	0.75	49.31 ^a^	0.37	3.02	8.85	92.90 ^a^
4	B6	83.80	1.14	5.25	0.17	1.32	6.41	42.76 ^a^
8		841.35 ^a^	8.72 ^a^	31.40 ^a^	0.24	4.10	59.16 ^a^	310.13 ^a^
4	B7	36.56	0.34	2.76	0.09 ^a^	0.69	4.44	7.05
8		41.02	0.31	4.36	0.31	2.12	5.02	20.32 ^a^
4	B8	28.54	0.28	1.06	0.01 ^a^	1.02	2.77	16.59
8		27.92	0.55	3.19	0.06 ^a^	0.48 ^a^	2.91	11.39
4	B9	29.89	0.29	1.60	0.04 ^a^	0.41 ^a^	8.85	36.99 ^a^
8		58.16	0.25	12.85 ^a^	ND	1.94	4.61	53.45 ^a^

^a^ Determinations yielding values below as well as above the linearity of the calibration curve (LOQ and upper LOQ, respectively); ND—not detected (below limit of detection, LOD).

**Table 7:** Statistical data on comparison of means between formulations with and without antioxidants (week 4, week 8). *t* and |*Z*| as critical values of the respective statistical test.

Odorant	Comparison of Means					
	*t*(16), |*Z*|		*Significance* (*p* < 0.05)		*p*-Value	
	Week 4	Week 8	Week 4	Week 8	Week 4	Week 8
hexanal	−5.48 ^b^	−3.00 ^c^	sig.	sig.	2.98 × 10^−4^	2.68 × 10^−3^
(*E*)-hex-2-enal	−3.77 ^b^	−2.91 ^c^	sig.	sig.	0.01	3.57 × 10^−3^
oct-1-en-3-one	−0.61 ^a^	−2.62 ^b^	n.s.	sig.	0.55	0.03
(*Z*)-octa-1,5-dien-3-one	−1.52 ^a^	−2.41 ^b^	n.s.	sig.	0.15	0.04
(*E*,*Z*)-nona-2,6-dienal	−3.39 ^b^	−2.07 ^c^	sig.	sig.	0.01	0.04
(*E*,*E*)-deca-2,4-dienal	−2.08 ^b^	−2.30 ^c^	n.s.	sig.	0.05	0.02
*trans*-4,5-epoxy-(*E*)-dec-2-enal	−2.91 ^c^	−2.30 ^c^	sig.	sig.	3.57 × 10^−3^	0.02

^a^ Student’s *t*-test; ^b^ Welch-test; ^c^ Mann-Whitney *U-* test.

**Table 8:** Statistical data on one-way repeated ANOVA combined with posthoc-test ^1,2^ for pairwise comparison of the means: analysis of lipid oxidation via secondary lipid oxidation products over storage time.

Odorant	Formulation	Repeated Measures ANOVA		Post-hoc Test Week 0–4		Post-hoc Test Week 0–8	
		*F*-Value	*p*-Value	*p*-Value	Significance (*p* < 0.05)	*p*-Value	Significance (*p* < 0.05)
hexanal	A7	5.21	0.08	0.98 ^1^	n.s.	0.04 ^2^	sig.
	B4	11.45	0.02	0.78 ^1^	n.s.	0.01 ^2^	sig.
	B6	6.51	0.06	0.98 ^1^	n.s.	0.03 ^2^	sig.
(*E*)-hex-2-enal	A7	1.36	0.42 *	-	-	-	-
	B4	8.37	0.04	0.21 ^2^	n.s.	0.02 ^2^	sig.
	B6	14.50	0.01	0.90 ^1^	n.s.	0.01 ^2^	sig.
oct-1-en-3-one	A7	5.39	0.07	0.56 ^1^	n.s.	0.07 ^1^	n.s.
	B4	213.20	8.64 × 10^−5^	0.10 ^1^	n.s.	4.41 × 10^−5^^2^	sig.
	B6	3.22	0.15 *	-	-	-	-
(*Z*)-octa-1,5-dien-3-one	A7	1.05	0.43 *	-	-	-	-
	B4	41.54	2.11 × 10^−3^	0.11 ^1^	n.s.	8.85 × 10^−4^^2^	sig.
	B6	140.39	1.97 × 10^−4^	8.52 × 10^−4^^1^	sig.	8.02 × 10^−5^^2^	sig.
(*E*,*Z*)-nona-2,6-dienal	A7	8.30	0.11 *	-	-	-	-
	B4	91.40	4.59 × 10^−4^	0.05^1^	n.s.	4.41 × 10^−4^^1^	sig.
	B6	46.51	1.70 × 10^−3^	0.32^1^	n.s.	1.82 × 10^−3^^1^	sig.
(*E*,*E*)-deca-2,4-dienal	A7	4.20	0.10 *	-	-	-	-
	B4	12.65	0.02	0.82 ^1^	n.s.	0.02 ^1^	sig.
	B6	35.55	2.84 × 10^−3^	0.89 ^1^	n.s.	1.67 × 10^−3^^2^	sig.
*trans*-4,5-epoxy-(*E*)-dec-2-enal	A7	0.38	0.73 *	-	-	-	-
	B4	22.22	0.01	0.15 ^1^	n.s.	2.74 × 10^−3^^2^	sig.
	B6	10.11	0.03	0.69 ^2^	n.s.	0.01 ^2^	sig.

* no post-hoc test necessary (*p* ≥ 0.10, n.s.); ^1^ homogeneity of variance: Tukey test; ^2^ non-homogeneity of variance: Fisher LSD test.

### 3.2. Monitoring of Autoxidation—Analysis of Lipid Hydroperoxides and Conjugated Dienes

Two of the standard tools for monitoring fatty acid oxidation involve the determination of lipid hydroperoxides (LPO) and of conjugated dienes. As mentioned in Section 1, these methods focus on the formation of primary lipid oxidation products, which can be measured photometrically. Here, both lipid hydroperoxides and conjugated dienes were monitored in all 18 infant formulas over a storage period of 12 months. The results are shown in Table 9 and Table 10. LPOs as well as conjugated dienes were detectable in all formulations using the methods described in Section 2.7.

Table 9 shows that evaluation of the process of lipid oxidation via thiocyanate assay by monitoring of the formation of LPOs was very limited directly after production, as well as during the first weeks of storage. LPOs were first quantifiable at concentrations above the LOQ (here: 1 mmol·kg^−1^) in formulations without antioxidants (B-formulations) after eight weeks of storage, with the exception of formulations B7 and B8. For A-formulations with antioxidants (except A7–A9), LPOs were detectable at concentrations above the LOQ after three months of storage. In general, formulations B7, B8 and A7–A9 exhibited lower concentration levels over a storage time of 12 months (<5 mmol·kg^−1^ for A7–A9, <50 mmol·kg^−1^ for B7 and B8). These findings are in agreement with the results of the 2D-HRGC-MS/O analysis (Section 3.1).

For the formation of conjugated dienes (Table 10), relatively constant concentrations from 10 to 20 mmol·kg^−1^ were detected over the first three months of storage. An initial increase in concentration was found for B-formulations after six months and A-formulations after nine months with concentrations above 20 mmol·kg^−1^. After nine months of storage only the formulations A4, A7–A9, B7 and B8 showed conjugated diene concentrations below 20 mmol·kg^−1^. These results again confirm that both stable and less stable formulas could be found among all 18 formulations investigated. Again, formulations A7–A9, B7 and B8 were the most stable formulations, indicating that encapsulated Cu^2+^ seems to inhibit lipid autoxidation independent from the presence of (non-)encapsulated iron.

**Figure 3:**
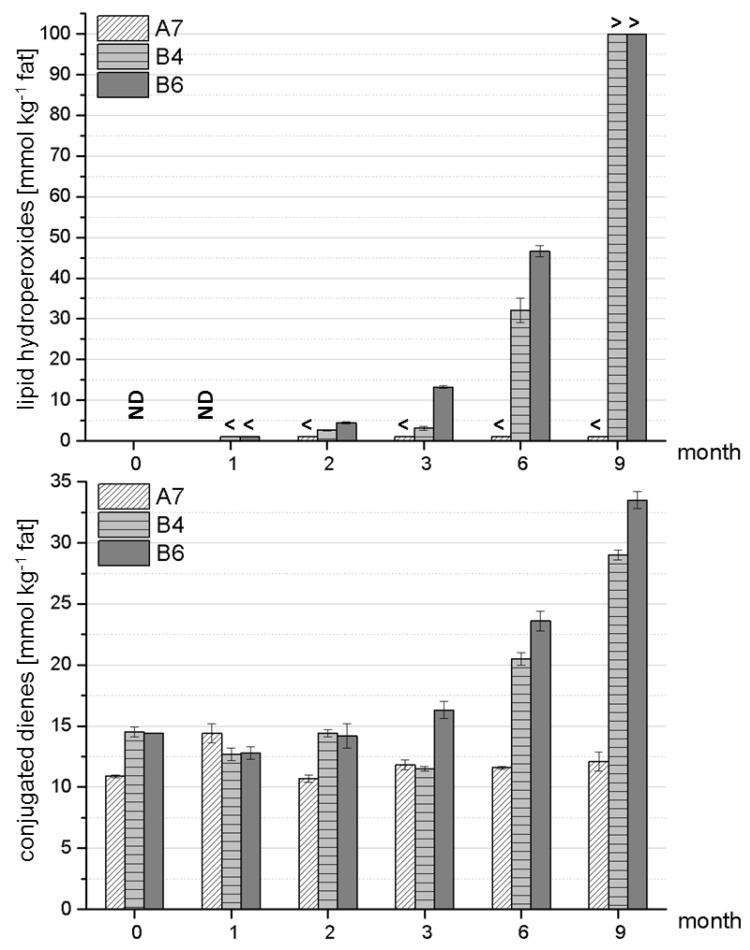
Example of a stable formulation (A7) compared to two less stable formulations (B4, B6) with respect to lipid autoxidation/formation of primary lipid oxidation markers. Analyses were performed in triplicate on two independent reconstitutions of powdered formulas. ND—not detected (below LOD); < below LOQ; > upper LOQ exceeded.

In general, it seems that the process of oxidative deterioration can be evaluated by means of the formation of LPOs and conjugated dienes as primary markers of fatty acid oxidation. Nevertheless, comparably long storage intervals were necessary in the present study (when samples are stored under vacuum) to confirm differences in the stability of the formulas. To confirm the latter statement, an ANOVA was carried out on the obtained data. Figure 3 displays the formation of LPOs and conjugated dienes over nine months of storage. As in Section 3.1, the formulations A7, B4 and B6 were chosen as representative samples for stable (A7) and less stable (B4, B6) formulations. Details on the respective statistical data for these formulations are shown in Table 11 for the formation of LPOs and Table 12 for the formation of conjugated dienes. In the case of LPOs, storage data was compared to month 1 since all data of month 0 was below the limit of quantification (LOQ). Due to outliers (*cf.* Section 2.9), month 1 was not included in the one-way repeated ANOVA on conjugated dienes.

**Table 9:** Analysis of lipid hydroperoxides in infant formulations with and without antioxidants via thiocyanate assay.

Formulation	Concentration (mmol·kg^−1^)													
	Month 0		Month 1		Month 2		Month 3		Month 6		Month 9		Month 12	
	Mean	SD	Mean	SD	Mean	SD	Mean	SD	Mean	SD	Mean	SD	Mean	SD
A1	ND	-	<1.0	-	<1.0	-	1.3	±0.2	17.8	±0.3	49.9	±1.6	NA	-
A2	ND	-	ND	-	1.6	±0.2	11.1	±0.6	42.5	±1.4	>100.0	-	NA	-
A3	ND	-	ND	-	1.2	±0.5	7.6	±1.1	38.4	±2.4	>100.0	-	NA	-
A4	<1.0	-	ND	-	<1.0	-	1.6	±0.1	11.2	±0.3	32.2	±2.4	NA	-
A5	<1.0	-	ND	-	<1.0	-	11.1	±1.0	20.0	±1.3	54.4	±1.9	NA	-
A6	<1.0	-	ND	-	<1.0	-	2.3	±0.1	15.0	±0.8	44.3	±0.8	NA	-
A7	ND	-	ND	-	<1.0	-	<1.0	-	<1.0	-	<1.0	-	2.9	±0.9
A8	<1.0	-	ND	-	<1.0	-	<1.0	-	<1.0	-	<1.0	-	2.6	±0.6
A9	<1.0	-	ND	-	<1.0	-	<1.0	-	<1.0	-	1.2	±0.4	1.2	±0.8
B1	<1.0	-	<1.0	-	1.4	±0.3	3.6	±0.3	18.2	±0.4	45.6	±1.6	NA	-
B2	ND	-	<1.0	-	2.2	±0.1	5.0	±0.3	20.9	±0.8	54.5	±1.9	NA	-
B3	ND	-	<1.0	-	4.1	±0.2	1.9	±0.4	40.4	±1.9	>100.0	-	NA	-
B4	ND	-	<1.0	-	2.6	±0.1	3.1	±0.5	32.1	±3.0	>100.0	-	NA	-
B5	ND	-	<1.0	-	3.3	±0.2	8.2	±1.0	32.6	±1.3	>100.0	-	NA	-
B6	ND	-	<1.0	-	4.5	±0.2	13.2	±0.3	46.6	±1.4	>100.0	-	NA	-
B7	ND	-	<1.0	-	<1.0	-	2.5	±0.2	7.3	±0.4	17.5	±0.9	48.0	±2.2
B8	<1.0	-	<1.0	-	<1.0	-	1.1	±0.0	4.0	±0.3	15.1	±1.7	40.3	±1.7
B9	ND	-	<1.0	-	1.6	±0.1	2.8	±0.1	12.5	±0.2	40.8	±2.9	NA	-

Analyses were performed in triplicate on two independent reconstitutions of powdered formulas. ND—not detected (below LOD); NA—not analyzed (since upper LOQ was exceeded at previous point of investigation).

**Table 10:** Analysis of conjugated dienes in infant formulations with and without antioxidants via UV absorption.

Formulation	Concentration (mmol·kg^−1^)													
	Month 0		Month 1		Month 2		Month 3		Month 6		Month 9		Month 12	
	Mean	SD	Mean	SD	Mean	SD	Mean	SD	Mean	SD	Mean	SD	Mean	SD
A1	12.9	±0.5	13.3	±1.0	14.4	±1.5	10.8 *	±1.6	15.8	±0.5	21.9	±1.5	NA	-
A2	11.0	±0.2	13.7	±0.4	11.9 *	±0.4	16.5	±0.4	19.0	±0.4	30.9	±0.7	NA	-
A3	11.0	±0.1	13.4	±0.8	11.6 *	±0.2	15.7	±0.3	18.2	±0.5	29.9	±0.8	NA	-
A4	11.1	±0.2	11.8	±0.7	10.2 *	±0.4	11.0	±0.2	14.6	±0.2	19.0	±1.0	NA	-
A5	11.3	±0.0	10.4	±1.2	10.8	±0.5	15.7	±0.2	16.5	±0.4	24.5	±1.0	NA	-
A6	12.5	±1.2	13.1	±0.5	10.6	±0.2	11.3	±0.4	15.7	±0.3	22.8	±0.4	NA	-
A7	10.9	±0.1	14.4 *	±0.8	10.7	±0.3	11.8	±0.4	11.6	±0.1	12.1	±0.8	11.7	±0.2
A8	11.2	±0.2	13.5 *	±0.6	10.9	±0.4	11.5	±0.5	11.8	±0.3	13.1	±0.7	11.7	±0.3
A9	10.9	±0.5	11.5	±0.8	10.4	±0.2	10.7	±0.2	11.1	±0.1	13.0	±0.2	11.3 *	±0.6
B1	13.7	±0.2	14.4	±0.5	14.4	±2.0	14.7	±0.6	16.9	±0.5	23.1	±0.7	NA	-
B2	13.9	±0.1	14.3	±0.1	13.5	±0.2	14.0	±0.4	18.7	±0.9	25.6	±0.6	NA	-
B3	12.5	±0.6	14.4	±0.5	14.4	±0.2	13.8 *	±0.2	22.1	±0.7	31.2	±0.8	NA	-
B4	14.5	±0.4	12.7 *	±0.5	14.4	±0.3	11.5 *	±0.2	20.5	±0.5	29.0	±0.4	NA	-
B5	15.1	±1.8	11.1 *	±5.1	14.6	±0.4	15.6	±0.6	21.2	±0.2	29.0	±0.9	NA	-
B6	14.4	±0.0	12.8 *	±0.5	14.5	±0.9	16.3	±0.7	23.6	±0.8	33.5	±0.7	NA	-
B7	12.5	±0.3	13.7	±0.2	14.0	±0.4	11.6 *	±0.1	14.4	±0.5	16.8	±1.0	22.2	±0.5
B8	11.6	±1.0	12.9	±0.6	14.0	±0.4	10.3 *	±0.7	13.9	±0.3	16.5	±0.7	21.8	±0.3
B9	14.0	±0.2	12.9 *	±0.4	14.1	±0.4	13.5	±0.2	16.2	±1.2	23.2	±1.2	NA	-

Analyses were performed in triplicate on two independent reconstitutions of powdered formulas. NA—not analyzed (*cf.* Table 9); * outlier (*cf.* Section 2.9).

**Table 11:** Statistical data on one-way repeated ANOVA combined with a post-hoc test ^1,2^ for pairwise comparison of the means: analysis of lipid oxidation via lipid hydroperoxides over storage period.

Odorant	Formulation	Repeated Measures ANOVA		Post-hoc Test Month 1–2		Post-hoc Test Month 1–3		Post-hoc Test Month 1–6		Post-hoc Test Month 1–9	
		*F*-Value	*p*-Value	*p*-Value	Significance (*p* < 0.05)	*p*-Value	Significance (*p* < 0.05)	*p*-value	Significance (*p* < 0.05)	*p*-Value	Significance (*p* < 0.05)
Lipid hydroperoxides	A7	- *	- *	-	-	-	-	-	-	-	-
	B4	2142.26	5.14 × 10^−26^	0.99^1^	n.s.	0.56 ^2^	n.s.	1.85 × 10^−6^^2^	sig.	0.00 ^2^	sig.
	B6	712.14	2.97 × 10^−21^	1.00^1^	n.s.	0.32 ^2^	n.s.	1.10 × 10^−3^^2^	sig.	0.00 ^2^	sig.

^1^ homogeneity of variance: Tukey test; ^2^ non-homogeneity of variance: Fisher LSD test; * insufficient data for ANOVA (month 1: below LOD).

**Table 12:** Statistical data on one-way repeated ANOVA combined with a post-hoc test ^1,2^ for pairwise comparison of the means: analysis of lipid oxidation via conjugated dienes over storage period.

Odorant	Formulation	Repeated Measures ANOVA		Post-hoc Test Month 0–2		Post-hoc Test Month 0–3		Post-hoc Test Month 0–6		Post-hoc Test Month 0–9	
		*F*-Value	*p*-Value	*p*-Value	Significance (*p* < 0.05)	*p*-Value	Significance (*p* < 0.05)	*p*-Value	Significance (*p* < 0.05)	*p*-Value	Significance (*p* < 0.05)
Conjugated dienes	A7	15.53	6.30 × 10^−6^	0.98 ^1^	n.s.	1.90 × 10^−3^^2^	sig.	0.02 ^1^	sig.	9.87 × 10^−5^^2^	sig.
	B4	2357.51	1.98 × 10^−26^	0.99 ^1^	n.s.	2.10 × 10^−7^^1,^*	sig. *	0.00 ^1^	sig.	0.00 ^1^	sig.
	B6	788.05	1.09 × 10^−21^	0.66 ^2^	n.s.	1.63 × 10^−4^^2^	sig.	1.78 × 10^−15^^2^	sig.	0.00 ^2^	sig.

^1^ homogeneity of variance: Tukey test; ^2^ non-homogeneity of variance: Fisher LSD test; * outlier.

The findings reported in Table 11 and Table 12 confirm that it was possible to detect significant differences in the stability of the formulas via the photometrical detection of LPOs and conjugated dienes. However, statistical significance of the data was achieved only with storage period intervals of at least six months for LPOs and three to six months for conjugated dienes. Hence, neither method was sufficiently sensitive in the current case to identify and monitor the emergence of oxidative lipid deterioration processes in its very early stages.

To complete the data analyses performed in Section 3.1, A-formulations were compared with B-formulations to reveal if the measurements of primary products of lipid oxidation via the presented methods were successful with regard to detection of significant differences between formulations with and without antioxidants. In the case of the thiocyanate assays (formation of LPOs), no significances could be found over storage period [*t*(16) = −0.19373, 1.03466, −1.18395, *p* < 0.05 (n.s.) (3, 6 and 9 months)]. Data from week 0 to 8 were not investigated with regard to significances, since concentrations in most of the samples were below the LOQ of the method.

With respect to conjugated dienes, a significant difference was found between A-formulations and B-formulations directly after production, as well as after a storage time of eight weeks and six months [*t*(16) = −4.608 (sig.), −0.818 (n.s.), −6.532 (sig., Welch), −0.668 (n.s.), −2.454 (sig.), −1.460 (n.s.), *p* < 0.05 (0, 1, 2, 3, 6 and 9 months)]. The occurrence of these differences between A- and B-formulations was not constant over the storage period, since differences between week 4, month 3 and month 9 were not significant.

### 3.3. Sensory Characterization of Infant Formulas during Storage—Aroma Profile Analysis

Samples of each formulation of reconstituted milk were evaluated by APA. The following odor attributes were scored via orthonasal evaluation with regard to their intensities: cooked milk-like, fatty, green-grassy, metallic, fishy, oily, blood-like. Table 13 shows the mean values of the perceived odor intensities as rated by the panel. All attributes were generally perceived in all formulations but were ranked with considerably divergent intensities in the different formulations at different storage periods. The attributes fatty and cooked milk-like were more related to the original flavor profile of the fresh infant formulas, as can be deduced from Table 13. For most formulas, the latter two attributes were ranked in the freshly produced samples as being clearly perceivable (odor intensities between 1.0 and 2.0). Also the attribute metallic was ranked with intensities of between 1 and 2 for most formulations. Nevertheless, all other odor attributes were evaluated in the freshly produced formulations with very low intensity (below 1.0), with only very few exceptions.

**Table 13:** Intensity rating of specific orthonasally perceived odor attributes in aroma profile analysis (APA) of reconstituted infant formulations with and without antioxidants. Mean values of evaluations by trained panelists (*n*_panelists_ = 8-12).

Storage (Week)	Intensity of Odor Quality (Scale 0–3, Mean Values)																	
	With Antioxidants									Without Antioxidants								
	Formulation	Fishy	Metallic	Oily	Green, Grassy	Blood-Like	Fatty	Cooked Milk-Like	Off-Flavor	Formulation	Fishy	Metallic	Oily	Green, Grassy	Blood-Like	Fatty	Cooked Milk-Like	Off-Flavor
0	A1	0.90	1.40	0.65	0.95	0.80	1.50	1.35	-	B1	1.06	2.28	0.89	1.06	0.44	1.39	1.33	*
4		0.61	1.33	0.78	1.22	0.44	2.00	1.83	-		1.28	1.78	0.72	1.00	1.00	1.17	0.72	*
8		0.78	1.33	0.50	1.00	0.72	1.22	1.56	*		1.20	2.00	0.95	1.00	0.90	1.25	1.05	*
24		1.17	2.17	1.75	1.42	1.42	1.42	1.08	*		0.96	2.08	1.08	1.13	1.21	1.38	1.25	-
0	A2	0.69	1.56	0.63	1.00	1.06	1.13	0.81	-	B2	0.33	0.61	0.39	0.89	0.11	1.44	2.11	-
4		1.32	1.82	1.09	0.91	1.09	1.27	1.45	*		0.89	1.28	0.61	0.50	0.72	0.94	1.22	-
8		1.22	1.83	1.28	1.11	1.33	1.56	0.72	*		1.30	2.40	1.00	0.95	1.15	1.30	1.20	*
24		0.68	2.23	1.05	1.09	1.45	0.82	1.05	*		0.86	1.95	1.00	1.00	1.27	0.91	1.18	*
0	A3	1.19	1.94	1.06	0.81	1.63	0.94	0.38	*	B3	0.91	1.50	0.91	0.82	1.00	0.86	1.27	-
4		1.18	1.86	0.91	1.14	0.95	1.23	1.18	*		0.70	1.64	0.80	0.95	1.09	1.09	1.50	*
8		1.39	2.11	1.06	0.72	1.39	1.17	1.00	*		1.27	2.09	1.05	0.77	1.55	1.05	0.86	*
24		0.95	1.82	1.14	0.86	1.14	1.05	0.77	-		1.36	2.14	1.36	1.09	1.27	1.14	0.68	-
0	A4	0.44	0.88	0.25	1.00	0.50	0.75	1.44	-	B4	0.60	0.90	0.55	0.55	0.60	1.05	1.55	*
4		0.44	0.90	0.39	0.61	0.28	1.17	1.94	-		1.11	1.94	1.11	0.56	1.00	1.17	1.28	*
8		1.35	1.65	1.30	0.75	1.25	1.20	1.25	*		0.83	2.28	0.50	0.89	1.28	1.00	1.50	*
24		0.77	1.91	0.82	0.73	1.27	0.95	1.18	*		1.21	2.04	1.63	1.21	1.42	1.54	0.54	*
0	A5	0.86	2.05	1.09	1.05	1.00	0.91	1.23	-	B5	0.45	0.95	0.35	0.70	0.55	0.85	1.05	-
4		0.60	1.73	0.55	0.40	1.10	0.95	1.14	-		0.72	1.72	0.72	0.94	1.17	1.06	1.06	-
8		0.94	1.50	1.21	0.85	1.50	0.90	1.50	*		1.00	2.44	0.67	1.17	1.83	0.67	0.89	*
24		0.79	2.25	1.63	1.50	1.50	1.50	0.96	*		1.54	2.13	2.04	1.42	1.58	1.29	0.71	*
0	A6	0.69	1.38	0.56	1.00	0.88	0.44	0.50		B6	0.65	0.95	0.40	0.15	0.55	0.70	1.10	-
4		0.67	1.67	0.56	0.83	0.83	1.00	1.39	*		0.72	1.50	1.00	0.50	1.11	1.22	1.17	*
8		1.15	1.50	1.05	0.65	1.30	1.20	0.85	*		1.22	2.44	1.17	0.83	1.94	1.00	1.06	*
24		0.64	2.14	1.00	1.05	1.18	1.05	1.23	*		1.83	2.25	1.88	1.21	1.46	1.04	0.92	*
0	A7	0.44	1.31	0.31	0.88	0.81	0.88	1.38	-	B7	0.91	1.18	0.85	0.82	0.68	1.14	1.32	-
4		0.77	1.00	0.68	0.82	0.64	1.23	1.68	-		0.50	0.70	0.44	0.40	0.80	1.14	1.82	-
8		0.61	1.28	0.67	0.56	0.89	1.33	1.67	-		0.68	1.64	0.91	0.59	1.05	0.95	1.45	*
24		0.29	1.25	0.54	0.83	1.25	1.50	1.67	-		1.46	1.75	1.63	1.25	1.42	1.33	1.04	*
0	A8	0.56	1.31	0.81	0.88	1.00	0.69	0.75	-	B8	0.73	1.18	0.95	0.68	0.82	0.86	1.05	-
4		0.06	0.78	0.06	0.72	0.06	0.67	1.22	-		0.11	0.83	0.17	0.20	0.67	0.82	1.41	-
8		0.83	1.22	0.56	0.56	0.94	1.00	1.00	-		0.50	1.27	0.64	0.36	0.82	1.00	1.55	-
24		0.58	1.29	0.58	0.79	1.38	1.63	1.75	-		0.41	1.14	0.50	0.45	0.77	1.27	1.55	-
0	A9	0.56	0.63	0.56	0.81	0.69	1.56	1.50	-	B9	0.75	1.40	0.80	0.85	0.90	1.05	1.10	-
4		1.00	2.17	0.72	1.06	0.89	0.83	1.67	*		1.17	2.00	0.72	0.56	1.00	1.00	1.06	*
8		1.65	1.75	1.40	0.75	1.35	1.25	1.20	*		0.94	2.00	0.67	0.78	1.00	0.56	1.22	*
24		0.71	1.38	0.25	0.71	1.00	1.42	2.04	-		1.67	2.33	1.92	0.96	1.83	1.17	0.88	*

* more than 2/3 of the panelists agreed on the presence of an off-flavor.

**Figure 4:**
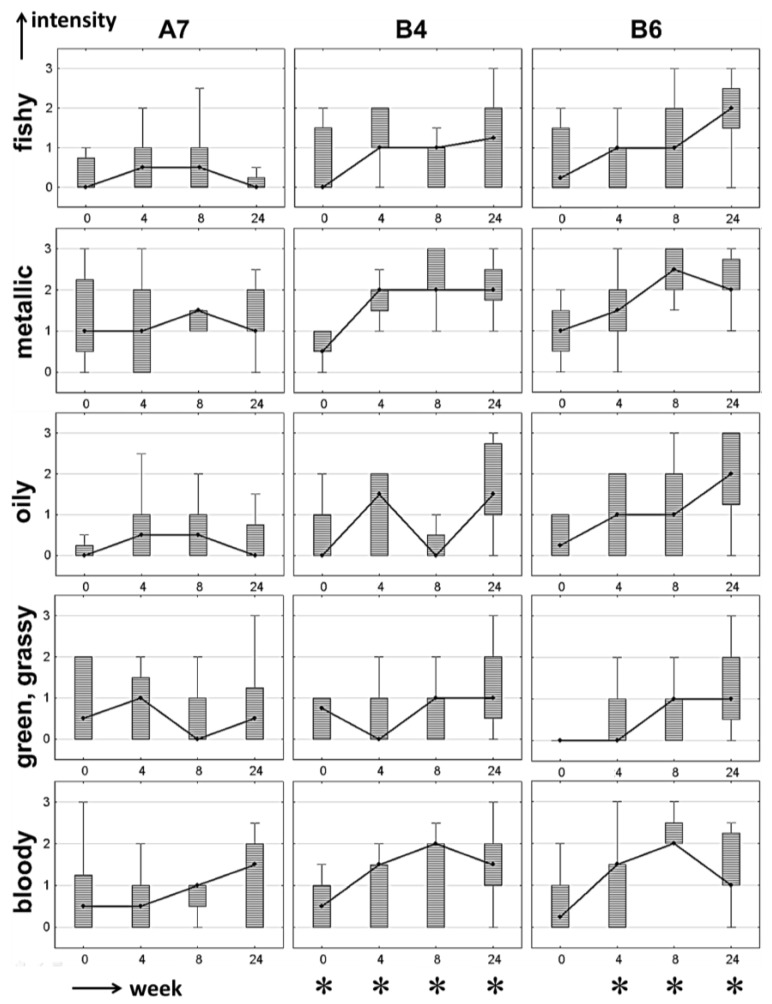
Box-plots of sensory evaluation of a stable formulation (A7) and two less stable formulations (B4, B6) over storage period (weeks). *Median*: **•**; *whiskers*: ±minimum-maximum ratings (without outliers); *box*: percentiles 25%–75%. * off-flavor (perceived by more than 2/3 of the panelists).

The sensory profile of the infant formulas changed with storage period and attributes like metallic, green-grassy, blood-like, oily and fishy became increasingly perceivable. Based on off-flavors determined via APA, only a few formulations remained stable over the entire storage period. It was shown in Section 3.1 and Section 3.2 that formulation A7 (among others) was very stable in terms of the formation of primary and secondary lipid oxidation markers. Formulations B4 and B6 were less stable, as displayed in Figure 2 and Figure 3. To demonstrate changes in the sensory profile over storage period, these three formulations are discussed here again as representative samples to show the results of the APA in more detail. Thus, the data is represented as box-plot diagrams for five of the seven odor attributes (Figure 4). Instead of the arithmetic mean, median values are plotted due to their robustness towards outliers.

In the case of formulation A7, all off-odor related odor attributes listed in Figure 4 were rated as being very low in odor intensity, with median values between not perceivable (0) and weakly perceivable (1) in most cases. Only the odor attribute cooked milk-like (Table 13) was rated as clearly perceivable, with intensity values greater than 1.5 from a storage period of four weeks upwards. Accordingly, the results from the quantification experiments revealing A7 as a very stable formulation were confirmed by APA. For formulations B4 and B6, odor intensities were also rated as being very low for week 0 samples. The APA of week 4 revealed increasing median values for the metallic and blood-like attributes, with high ranking for both parameters also in week 8 and 24 (*cf.* Figure 4). For the odor qualities fishy, oily and green-grassy, the same trend towards a steadily increasing ranking with storage period was observed, especially when focusing on the 25%–75% quartiles of the box-plots. These results are in good correlation with the quantification results from Section 3.1 and Section 3.2, respectively.

Nevertheless, even if a number of off-flavor related odor attributes were ranked as being well perceivable and, in general, correlated well with the quantitative data, an APA does not directly reveal if the overall odor profile is still acceptable or not. Furthermore, the overall flavor profile may change from one dominating attribute to another (e.g., metallic to fishy) and thus, specific attributes may decrease over time even if an overall off-flavor is arising or intensifying, as can be seen in Figure 4 in the case of the attribute blood-like in formulations B4 and B6 after six months (week 24). In these samples, the odor profile changed such that attributes like fishy and oily were perceived as being stronger (presumably because these attributes are more closely related to an off-flavor). Thus, the sensory panel was additionally asked to evaluate if the sample was related to an overall off-flavor or not (indicated with an asterisk in Figure 4 when more than 2/3 of the panelists agreed on the presence of an off-flavor).

For a better overview, Table 13 also includes a column indicating where an off-flavor was reported in the formulations. As can be seen from Table 13, neither formulation A7, A8 nor B8 were rated as having an off-flavor throughout the storage period. These observations are consistent with the results of the APA and the analyses reported in Section 3.1 and Section 3.2 on the stability of the different infant formulas in terms of oxidative deterioration. Nevertheless, comparison of the off-flavor rankings, the APA results, and the results of the quantification experiments are not compatible in some minor cases. One example shall be given for the odor attribute metallic and the quantitative data obtained for *trans*-4,5-epoxy-(*E*)-dec-2-enal, an odor-active compound with a metallic flavor [5]. For most formulations over the storage period there is a good correlation of sensory and quantitative experiments, except for formulations A5 and B5 after four weeks of storage. In the latter example the panelists did not agree on the presence of an off-flavor, even if the concentration of *trans*-4,5-epoxy-(*E*)-dec-2-enal was detected in formulation B5 at a concentration that was 18 times higher than in formulation A5. At the same time, the panelists performing the APA rated the odor attribute *metallic* as being clearly perceivable and comparable in both samples (APA rating 1.73 *vs.* 1.72 for formulations B5 and A5, respectively). This is a very good example to demonstrate that an off-flavor (here: metallic) is perceivable and the concentrations of the volatiles responsible for this off-odor [here: *trans*-4,5-epoxy-(*E*)-dec-2-enal] are elevated, but the overall flavor profile is a mixture resulting from the concentration ratio of alkanals, alkenals, alkadienals, alkenones and alkadienones. At a certain (unknown) point, when concentration ratios change due to on-going autoxidation, the overall flavor profile will also change, presumably from metallic towards fishy, oily or blood-like, respectively. 

In this context, there is another interesting finding from the data of sensory evaluation and off-flavor ranking: a metallic off-flavor seems to be more accepted by the panelists in the case of infant formulas. However, this acceptance changes to rejection if the concentration ratio of the volatiles changes into a fishy, oily or blood-like flavor note, as can be seen for all formulations: as soon as the intensity rating for fishy, oily or blood-like exceeded being well-perceivable (APA rating 1.5 or higher), the panelists agreed on the presence of an off-flavor, indicating that also consumers would reject these products.

Thus, it can be summarized that powdered infant formulas were found to develop fishy, oily or blood-like flavor notes that arise from a mixture of saturated and unsaturated aldehydes and ketones and impart an off-flavor to those products. A few formulations—namely A7, A8 and B8—were found to be very stable over the whole storage period with respect to quantitative experiments as well as sensory evaluations. Thus, it can be assumed that a combination of encapsulated Cu^2+^ and non-encapsulated Fe^3+^ is indeed a favorable combination to manufacture stable infant formulas with respect to their odor profile. 

In addition to APA, an instrumental color analysis was carried out over storage time to check any browning developments due to interactions between lipid oxidation products with proteins or polymerization of lipid oxidation products. No significant color changes could be detected by means of a paired samples *t*-test (week 0 *vs.* week 8), which showed the following results: L* *t*(17) = 1.368, *p* = 0.19 (n.s.), a* *t*(17) = −0.718, *p* = 0.48 (n.s.) and b* *t*(17) = −1.807, *p* = 0.09 (n.s.), respectively.

Overall, it was found that there was a very good correlation between the sensory profile, the off-flavor ranking, and the concentration of primary and secondary oxidation markers. Furthermore, the results of a multi-dimensional GC-MS analysis on odor-active aldehydes and ketones (*cf.* Section 3.1) demonstrated that it was possible to clearly distinguish formulations with and without antioxidants directly after production, even before a sensory panel could detect any obvious differences in the flavor profiles of the formulations. This finding is of enormous importance for quality monitoring and to hasten and optimize product developments of PUFA-enriched infant formulas, as well as of PUFA-enriched foods in general.

## 4. Discussion

Primary and secondary lipid oxidation markers were used as diagnostic tools in the process of oxidative deterioration of PUFA-enriched infant formulas during storage. The monitoring of volatiles from secondary fatty acid oxidation via GC-MS is described in the literature as a common operation tool to characterize fatty acid oxidation in foods [27,28]. The stability of the lipid fraction in infant formulas is frequently investigated via GC-MS. So far, the monitoring of the formation of saturated aldehydes such as propanal, pentanal, and hexanal has been the focus of many studies [16,18]. Nevertheless, the results of the present study show that it was possible to confirm further secondary oxidation markers besides the common alkanals: alkenals, alkadienals, alkenones and alkadienones, which were present at even lower concentrations but still with high odor potencies and described previously for UFA/conjugated linoleic acid (CLA)-enriched butter [27]. All volatiles investigated are extremely odor-active and thus of great sensorial importance for PUFA-enriched infant formulas and an early detection of on-going autoxidation processes. Moreover, it was possible by two-dimensional GC-MS measurements to clearly distinguish stable and less stable formulations directly after manufacturing and thus, even before a sensory panel could detect clear differences in the respective flavor profiles. Spitzer and Buettner [29] carried out a quantitative study on lipid autoxidation in human milk. All seven odorants investigated in the present study were similarly part of the investigation of human milk. Concentrations in human milk after six months storage at −19 °C were to a great extent comparable with the concentrations described in the present study for spray-dried infant formula milks. Both this and the aforementioned study were based on multi-dimensional GC-MS/O analyses: thus, this analytical approach has proved to be a very sensitive tool to determine lipid oxidation in milk matrices quickly after production—in the current case within the first weeks. These first weeks are of the utmost interest for the food industry in terms of quality control as well as to speed up product improvements. The results of quantification further confirmed that alkyl radicals (RO•) and peroxyl radicals (ROO•) were formed during production, leading to the formation of hydroperoxides (ROOH) in the presence of oxygen by a free radical chain mechanism [30,31]. Once these hydroperoxides are established, lipid oxidation may even proceed further under exclusion of oxygen, leading to increasing concentrations of secondary lipid oxidation products, as shown in Section 3.1. These findings are confirmed by the investigations of Chávez-Servín *et al.* [32] on packaged infant formulas.

When regarding previous studies on volatile oxidative degradation products, hexanal is reported to be one of the most common markers. Static headspace GC measurements have detected hexanal at concentrations in the low mg·kg^−1^ range (ppm); dynamic headspace sampling, purge-and-trap sampling, and classical liquid injection GC-MS measurements were found to be more sensitive (*ppb*-range) in terms of hexanal values [13,27,32,33]. Nevertheless, it should be noted that the present study—using a multi-dimensional approach—was able to even detect volatiles in the very low *ppb*-range [<0.1 μg·kg^−1^ for concentrations of (*E*)-hex-2-enal]. This is of great importance for detecting sensory changes in PUFA-enriched infant formulas from on-going autoxidation processes due to the very low odor thresholds of such compounds.

The formation of primary lipid oxidation markers via the photometrical methods presented in the current study also confirmed on-going autoxidation processes in the packaged, evacuated infant formulas even if longer time intervals were necessary to obtain significant results. Conjugated dienes provide a valuable tool to draw conclusions on the “thermal history” of oils as recently reported by Cihelkova *et al.* [34]. Geometrical isomerization of double bonds of fatty acids may occur during deodorization and refining of oils. Since hydroperoxides are degraded during these process steps and volatile compounds are removed, conjugated dienes are a reliable parameter for quality evaluation of the oils used [35]. A comprehensive review of the relevant literature reveals that the determination of peroxide value (PV) or hydroperoxides in infant formulas is generally not reported in the context of very early detection of oxidation processes. For instance, Chávez-Servín *et al.* [18,32] described a storage period of 70 days for infant formulas after the packaging was opened and 18 months in the case of packaged infant formulas for detection of these primary oxidation markers. Only Manglano *et al.* [36] found significant increases in PV and hydroperoxides during the first five months of storage of commercial vacuum-packed infant formulas. Therefore, the findings of the present study on sensitivity and consistency of methods for determination of primary oxidation products reflect the previous reports in the literature quite well. These methods have been described before as standard tools in industrial routine control for monitoring fatty acid oxidation in fats and oils as well as milk matrices [37,38] and are used in standard protocols for the determination of the hydroperoxide content in anhydrous milk fat [23]. Therefore, depending on the matrix under investigation, it might be useful to monitor both primary and secondary lipid oxidation products in samples where the oxidative status is unknown.

Having a closer look at the results of this study, it was shown that A-formulations with antioxidants remained more stable than B-formulations without antioxidants, which is in accordance with previous reports [30]. Again, statistical analyses revealed differences in the sensitivity of the different methods used, with 2D-HRGC-MS/O measurements of secondary lipid oxidation products providing the most reliable results directly after production and during the first weeks of storage. To the best of our knowledge, no other studies involving an experimental design in production of infant formulas with and without antioxidants and with additional monitoring of their oxidative as well as sensory stability have been reported in the literature.

Another outcome of this study is that an important technological aspect could be elaborated using the combinatory approach of analytical analyses and comparative evaluation of sensitivity: formulations 7 and 8 with encapsulated Cu(II)-salts and non-encapsulated Fe(II) and Fe(III)-salts appeared to be more stable than other mineral combinations of copper and iron. In general, transition metals such as copper and iron are described as key promotors of lipid oxidation in the aqueous phase of oil-in-water emulsions [30]. As in the present study, Manglano *et al.* [36] performed a systematic investigation on infant formulas, albeit just in relation to different Fe(II)-salts. All formulas were supplemented with antioxidants, thereby only differing in tocopherol source. An outcome of the study of Manglano *et al.* [36] was that it was not possible to detect significant differences within the infant formulas with respect to lipid oxidation. These results are quite consistent with the findings of the present study, indicating a greater influence of copper on fatty acid oxidation together with the presence of antioxidants than of iron.

Sensory evaluations were performed in the present study since the challenge in the development of *n*-3 and *n*-6 fatty acid enriched infant formulas is to meet the consumers’ expectations by avoiding aversive off-flavors. As already described by Spitzer *et al.* [5] for human milk, the formation of the odorants investigated in the present study are obviously closely related to the development of an off-flavor in PUFA-enriched milk samples. The corresponding off-flavor in human milk has previously been described by Spitzer *et al.* [5] as fishy, metallic, rancid and fatty; the same flavor attributes that were also detected in the present study. For infant formulas, there are hardly any reports in the literature regarding the formation of off-flavors. García-Martínez [39] determined the rancidity of an infant formula without any PUFA-enrichment, judged by a panel consisting of six untrained panelists. Chávez-Servín *et al.* [32] performed sensory evaluations by means of Duo-Trio and Pairs comparison tests where attributes such as “better smell”, “better flavor” or “more rancid flavor” were used. Others, like Hausner *et al.* [13] did not perform APA but suggested a correlation between the detected volatile compounds and typical (off-) flavor notes in heated milk from citations in the literature. To close this gap, the present study implemented description of the aroma profiles of 18 different infant formulas over storage period and evaluation of the samples with regard to a subjective off-flavor rating by the panelists. Overall, these findings fit in the majority of cases to the results obtained from the analyses of primary and secondary products of lipid oxidation. At this point it should be highlighted again that the two-dimensional GC-MS analysis on secondary oxidation products is even more sensitive than the sensory panel in distinguishing stable and less stable formulations directly after production. This finding is of huge importance for the industry in the context of quality monitoring and product development of PUFA-enriched milk-based products.

In addition, it was shown that specific sensory attributes from APA continuously increased but, in some cases, also decreased during storage (specifically when one dominating sensory impression changed to another). It was found that the overall flavor profile is dependent on a mixture of volatiles and resulting from specific concentration ratios of alkanals, alkenals, alkadienals, alkenones and alkadienones. At a certain point, when concentration ratios change due to an on-going autoxidation, the overall flavor profile will also change, e.g., from metallic towards a fishy, oily or a blood-like flavor. This phenomenon of the development of a fishy off-flavor has been described before for dry spinach [6]. Masanetz *et al.* [6] found that a shift in the concentration of (*Z*)-octa-1,5-dien-3-one and methional was responsible for a more dominant hay-like or fishy odor of dry spinach. Moreover, Venkateshwarlu *et al.* [7] investigated the influence of different alkenals, alkadienals, alkenones on the development of fishy and metallic off-flavors in pasteurized milk samples. A synergistic effect was found for (*E*,*Z*)-nona-2,6-dienal and (*Z*)-hept-4-enal with regard to a developing fishy off-flavor. In another study, Venkateshwarlu *et al.* [8] investigated fish oil-enriched milk and assumed that fishy and metallic off-flavors are the result of a combination of potent odorants that were identified in their study. The latter findings confirmed a high correlation of flavor changes in foods dependent on mixing ratios of specific volatiles.

Another aspect with high relevance to the present study on PUFA-enriched infant formulas is that Venkateshwarlu *et al.* [8] reported that GC/O analysis revealed no volatile compound(s) with a fishy flavor note. This finding is also in agreement with the GC/O results of the present study (results not shown).

With regard to the sensory evaluations and respective off-flavor rankings of the present study, it may be concluded that a dominant metallic flavor was found to be more acceptable for the panelists in the case of infant formulas than other off-odors that were generated over the course of product storage. This acceptance changed to a more aversive rating if the concentration ratio of the volatiles changed in a way that a fishy, oily or blood-like flavor note arose. To the best of our knowledge, no other studies have been reported that deal with the acceptance of panelists or consumers towards different (off-) flavors arising in PUFA-enriched infant formulas.

Thus, it can be concluded that it is not a mono-dimensional process to correlate specific volatile compounds with the formation of off-flavors. To understand the complexity of oxidative deterioration and off-flavor formation, the necessity of performing comprehensive sensorial investigations together with a detailed analytical elucidation of the underlying molecular processes could therefore be demonstrated with the present study, paving the way for future investigations in this important field of research. This study highlights the huge potential for technological improvements of infant formulas based on the applied combinatory approach.

## 5. Conclusions

According to McClements and Decker [30], progress in the development of fatty foods like infant formulas is strongly dependent on improved diagnostic methods to control and monitor their oxidative stability. The aim of this study was to monitor fatty acid oxidation in PUFA-enriched infant formulas in its early stages to support product development in terms of formula optimization. Thus, three different methods of monitoring lipid oxidation were applied in a comparative approach. All diagnostic tools of this study were found to be suitable with regard to confirmation of autoxidation processes under the present study conditions. Nevertheless, different statistical analyses revealed differences in the sensitivity of the different methods used, with respect to the results directly after production and during the first weeks of storage: 2D-HRGC-MS/O analysis of odorous marker substances was the most sensitive tool for early detection and monitoring of autoxidation in the milk matrices. This technique was even more sensitive than a sensory panel in differentiating stable and less stable formulations directly after production. Methods determining lipid hydroperoxides and conjugated dienes were suitable to indicate oxidative changes, albeit after sensory changes became apparent.

In addition, the composition of all infant formulas was varied with regard to antioxidants and the minerals iron and copper in the frame of an experimental design. By combining the data from chemo-analytical investigations with the results from sensory examinations it was shown that lipid oxidation was low when copper was administered in an encapsulated form and when anti-oxidants (vitamin E, ascorbyl palmitate) were additionally present. Accordingly, such findings offer the possibility to predict oxidative processes and to establish optimization strategies for development of infant formulas.

## Abbreviations

2D-HRGC-MS/O: two-dimensional high resolution gas chromatography-mass spectrometry/olfactometry
AA: arachidonic acid
ANOVA: analysis of variance
APA: aroma profile analysis
CLA: conjugated linoleic acid
DHA: docosahexaenoic acid
FID: flame ionization detector
FTIR: fourier transform infrared
GC: gas chromatography
GOS/FOS: galacto oligosaccharides/fructo oligosaccharides
HPLC: high performance liquid chromatography
IDF: international dairy federation
LC: long chain
LOD: limit of detection
LOQ: limit of quantification
LPO: lipid hydroperoxides
MS: mass spectrometry
ND/NA: not detected/not analyzed
n.s./sig.: not significant/significant
(P)UFA: (poly) unsaturated fatty acid
PV: peroxide value
SAFE: solvent assisted flavor evaporation
SD: standard deviation
SIDA: stable isotope dilution assay
UV: ultra violet
VOC: volatile organic compound
